# Patient-centred measurement in ophthalmology – a paradigm shift

**DOI:** 10.1186/1471-2415-6-25

**Published:** 2006-06-15

**Authors:** Konrad Pesudovs

**Affiliations:** 1 NHMRC Centre for Clinical Eye Research, Department of Ophthalmology, Flinders Medical Centre and Flinders University, Bedford Park, South Australia, 5042, Australia

## Abstract

Ophthalmologists and researchers in ophthalmology understand what a rapidly evolving field ophthalmology is, and that to conduct good research it is essential to use the latest and best methods. In outcomes research, one modern initiative has been to conduct holistic measurement of outcomes inclusive of the patient's point of view; patient-centred outcome. This, of course, means including a questionnaire. However, the irony of trying to improve outcomes research by being inclusive of many measures is that the researcher may not be expert in all measures used. Certainly, few people conducting outcomes research in ophthalmology would claim to be questionnaire experts. Most tend to be experts in their ophthalmic subspecialty and probably simply choose a popular questionnaire that appears to fit their needs and think little more about it. Perhaps, unlike our own field, we assume that the field of questionnaire research is relatively stable. This is far from the case. The measurement of patient-centred outcomes with questionnaires is a rapidly evolving field. Indeed, over the last few years a paradigm shift has occurred in patient-centred measurement.

## Text

Measurement of patient-centred health outcomes began in the 1950s, and more than 70 measures of functional status, 2 dozen generic quality of life instruments, and hundreds of disease-specific measures now exist [1-4]. Much of this research has relied on the theory and methods of Classical Test Theory (CTT). Underpinning CTT is simple scoring of responses to questionnaires; for multi-category response scales this is usually summary scoring where response categories are assigned ordinal numbers which are summed across questions to arrive at a total score. This score is assumed to represent measurement of the underlying trait (e.g. quality of life). An alternative approach is Item Response Theory (IRT) whereby items and persons can be scaled according to a series of responses to items. Implicit is that not only can people have different ability, but that items can have different difficulty and both can be estimated. The foundations of IRT were laid down in the 1920s, and great advances were made after 1950, especially with the contribution of Georg Rasch [5,6]. Rasch analysis is a special case of IRT similar to a one-parameter model, but importantly, Rasch models meet the conditions of non-interactive conjoint structures so, unlike IRT models, they are valid measurement models [7]. (See Massof's excellent background paper for further discussion of these methods as applied to ophthalmology [8]). It has only been in the last 25 years that Rasch analysis has been applied in studies of health status. Early applications included mental health and physical rehabilitation in the 1980s [9,10], including low vision rehabilitation [11,12]. Through the 1990s, and into the 21^st ^century, Rasch analysis has penetrated many health outcome fields and is becoming prevalent in ophthalmology.

However, many popular ophthalmic questionnaires use traditional summary scoring [13]. Summary scoring assumes the value of each item represents equal difficulty and therefore scores them equally. In addition, the ordinal integer response scale used for each item assumes uniform changes between response categories. For example, in a summary scored vision disability instrument such as the 'Activities of Daily Vision Scale (ADVS)'[14], a response of "a little difficulty" (score of 4) is used to represent twice the level of ability as "extreme difficulty" (score of 2) which is similarly two times as good as "unable to perform the activity due to vision" (score of 1) for all items. This appears illogical and Rasch analysis has been used to confirm that specific response category calibrations are required to provide a linear scale [15]. Similarly, summary scoring assumes that all items are of equal difficulty. For example, with the ADVS instrument an answer of "a little difficulty" to the question regarding visual difficulties 'driving at night' scores the same as "a little difficulty" with 'driving during the day'. Again, this is illogical and Rasch analysis has been used to confirm that subjects report that 'driving at night' is a more difficult task than 'driving during the day'; Rasch analysis can provide the appropriate weighting for each item to enable linear measurement [15].

By resolving inequities in a scale arising from differential item difficulty, Rasch analysis provides a self-evident benefit in terms of accuracy of scoring. However, this process also removes noise from the measurement which in turn improves sensitivity to change and correlations with other variables [16,17]. Clearly, these are important benefits for outcomes research with implications for sample sizes etc. The assumption of unidimensionality inherent in Rasch analysis also provides unparalleled insight into the dimensionality of a questionnaire. This can be used to advantage in questionnaire development. Therefore, beyond its simple application, Rasch analysis has been used in three important ways in ophthalmology: the development of new questionnaires, shortening or revising existing questionnaires, and test equating.

Across ophthalmology, a number of questionnaires have been developed using Rasch analysis. The field of low vision has led the way with the Veterans Affairs Low-Vision Visual Functioning Questionnaire, the Melbourne Low Vision ADL Index and the Visual Function Questionnaire [18-22]. Two Rasch scaled questionnaires have also been developed for refractive outcomes: one for spectacles, contact lenses and refractive surgery (The Quality of Life Impact of Refractive Correction, QIRC) and one for contact lenses only (Contact lens Impact on Quality of Life, CLIQ) [23-26]. While other sub-fields of ophthalmology are yet to gain questionnaires developed using Rasch analysis, these are likely not far away as a recent major review of quality of life in glaucoma called for the development of a Rasch scaled glaucoma-specific visual disability questionnaire [27].

Existing conventionally validated questionnaires can be rescaled using Rasch analysis. In this way, summary scoring can be converted to a truly linear scale using a simple formula. This has occurred for cataract surgery outcomes [28], refractive surgery outcomes [17], age-related macular degeneration outcomes [29], low vision care [30], and the measurement of ophthalmic pain [31,32]. Some of these approaches have been to simply rescore an existing questionnaire, while others have taken the approach of completely re-engineering a questionnaire to optimize its performance. The latter process takes advantage of the insight into dimensionality of a questionnaire to refine measurement and improve targeting of content to the population. Notable examples of questionnaire re-engineering are the Impact of Visual Impairment (IVI) for low vision outcomes [33,34], the Visual Disability Assessment (VDA) reinvented as the Cataract Outcomes Questionnaire [28,35], and The Visual Functioning 14 (VF-14) [36-38]. Usually, re-engineering of questionnaires involves removal of poorly fitting items to make a shorter questionnaire, although Velozo *et al *actually added items to the VF-14 and yet were unable to completely satisfactorily improve the instrument [36]. Rasch analysis has also been used to simply confirm the performance of, or detect deficiencies in questionnaires [39-42].

In ophthalmology, the most important patient-centred outcome measure is visual disability. Visual disability is the reason for performing cataract surgery, world wide the most common operation performed, and visual disability is a consequence of many ophthalmic diseases. Accordingly, there are many visual disability questionnaires e.g.: the Visual Activities Questionnaire (VAQ) [43], the Activities of Daily Vision Scale (ADVS) questionnaire [44], the VF-14 [45], the VDA [35], the National-Eye Institute Visual Functioning Questionnaire (NEI-VFQ)[46] and the Catquest questionnaire [47]. These questionnaires are widely used in different part of the world, for instance Catquest is widely used in Europe, the VF-14 in North America, and the VDA in Australia. While these questionnaires all measure the same concept, and have many of the same items, their scores cannot be simply compared. However, Rasch analysis provides a mechanism to equate scores from different questionnaires. Since all these questionnaires measure the same underlying trait, visual disability, they can all be modelled on the same latent variable. Massof has made an important first step in equating visual disability questionnaires implementing the ADVS, VAQ, VF-14 and the NEI-VFQ on a large population, conducting Rasch analysis and providing equations for conversion of summary scores to Rasch scores and conversion between questionnaires [48]. While Rasch analysis is in itself fairly simple, its application is somewhat specialized. Massof's equations provide a mechanism to gain the benefits of Rasch scaling without having to perform Rasch analysis.

Today, the outcomes researcher is faced with many different published questionnaires to choose from; some summary scored, others Rasch scaled. A major recent review by De Boer *et al *has attempted to make sense of this choice by systematically classifying questionnaires by the quality of their development and validation [49]. Notably, de Boer *et al *included Rasch scaling as a point of differentiation. A logical extension of this would be to rate highest questionnaires developed using Rasch analysis, followed by those conventionally developed and re-engineered using Rasch analysis and then those simply re-scored using Rasch analysis. There seems to be little advantage in using non-Rasch scaled questionnaires given this will increase noise and therefore reduce statistical power. Despite this, summary scored questionnaires remain popular [50], perhaps due to the simplicity of their scoring. But, many questionnaire developers, and other researchers, provide simple Rasch scale conversion obviating the need for questionnaire users to perform Rasch analysis [22,23,48]. This simple step would have added significant value to the paper by Owen *et al *published in this edition of BMC Ophthalmology [50]. Hopefully, others will agree that the time has come to abandon summary scoring. Lets just hope that Max Planck's pessimism about scientific change: "*a new scientific truth does not triumph by convincing its opponents and making them see the light, but rather because its opponents eventually die, and a new generation grows up that is familiar with it*" does not hold true for the Rasch analysis paradigm shift.

## Pre-publication history

The pre-publication history for this paper can be accessed here:


